# Socio-demographic correlates of unhealthy lifestyle in Ethiopia: a secondary analysis of a national survey

**DOI:** 10.1186/s12889-023-16436-7

**Published:** 2023-08-11

**Authors:** Yalemzewod Assefa Gelaw, Digsu N. Koye, Kefyalew Addis Alene, Kedir Y. Ahmed, Yibeltal Assefa, Daniel Asfaw Erku, Henok Getachew Tegegn, Azeb Gebresilassie Tesema, Berihun Megabiaw Zeleke, Yohannes Adama Melaku

**Affiliations:** 1 Telethon Kids Institute, Perth Children’s Hospital, Perth, WA Australia; 2 School of Population Health, Curtin University, Perth, WA Australia; 3 Centre for Epidemiology and Biostatistics, Melbourne School of Population and Global Health, The University of Melbourne, Melbourne, Victoria Australia; 4 Rural Health Research Institute, Charles Sturt University, Orange, NSW 2800 Australia; 5 Department of Public Health, Samara University, Samara, Afar Ethiopia; 6 School of Public Health, The University of Queensland, Brisbane, Australia; 7 Centre for Applied Health Economics, Griffith University, Nathan, QLD Australia; 8 Menzies Health Institute Queensland, Griffith University, Gold Coast, QLD Australia; 9 School of Rural Medicine, University of New England, Armidale, 2351 Australia; 10 Department of Clinical Pharmacy, School of Pharmacy, University of Gondar, Gondar, Ethiopia; 11 The George Institute for Global Health, University of New South Wales, Sydney, NSW Australia; 12 School of Population Health, University of New South Wales, Sydney, Australia; 13 Planetary Health Division, School of Public Health and Preventive Medicine, Monash University, Melbourne, Australia; 14 FHMRI Sleep Health, Flinders University, Adelaide, SA Australia; 15 Cancer Epidemiology Division, Cancer Council Victoria, Melbourne, Victoria Australia

**Keywords:** Socio-demographic, Lifestyle, NCDs, STEPS, Ethiopia

## Abstract

**Background:**

Multiple lifestyle risk factors exhibit a stronger association with non-communicable diseases (NCDs) compared to a single factor, emphasizing the necessity of considering them collectively. By integrating these major lifestyle risk factors, we can identify individuals with an overall unhealthy lifestyle, which facilitates the provision of targeted interventions for those at significant risk of NCDs. The aim of this study was to evaluate the socio-demographic correlates of unhealthy lifestyles among adolescents and adults in Ethiopia.

**Methods:**

A national cross-sectional survey, based on the World Health Organization's NCD STEPS instruments, was conducted in Ethiopia. The survey, carried out in 2015, involved a total of 9,800 participants aged between 15 and 69 years. Lifestyle health scores, ranging from 0 (most healthy) to 5 (most unhealthy), were derived considering factors such as daily fruit and vegetable consumption, smoking status, prevalence of overweight/obesity, alcohol intake, and levels of physical activity. An unhealthy lifestyle was defined as the co-occurrence of three or more unhealthy behaviors. To determine the association of socio-demographic factors with unhealthy lifestyles, multivariable logistic regression models were utilized, adjusting for metabolic factors, specifically diabetes and high blood pressure.

**Results:**

Approximately one in eight participants (16.7%) exhibited three or more unhealthy lifestyle behaviors, which included low fruit/vegetable consumption (98.2%), tobacco use (5.4%), excessive alcohol intake (15%), inadequate physical activity (66%), and obesity (2.3%). Factors such as male sex, urban residency, older age, being married or in a common-law relationship, and a higher income were associated with these unhealthy lifestyles. On the other hand, a higher educational status was associated with lower odds of these behaviors.

**Conclusion:**

In our analysis, we observed a higher prevalence of concurrent unhealthy lifestyles. Socio-demographic characteristics, such as sex, age, marital status, residence, income, and education, were found to correlate with individuals' lifestyles. Consequently, tailored interventions are imperative to mitigate the burden of unhealthy lifestyles in Ethiopia.

**Supplementary Information:**

The online version contains supplementary material available at 10.1186/s12889-023-16436-7.

## Introduction

Chronic diseases, including cardiovascular diseases, cancer, chronic respiratory diseases, and diabetes, are the most prevalent type of noncommunicable diseases (NCDs). These diseases are responsible for 80% of premature NCD-related deaths worldwide [1], leading to over 41 million deaths every year [2, 3]. Since 2005, the burden of NCDs has increased by almost 14% due to several factors, including changes in lifestyle and behavior, and increasing urbanization. Low-and middle-income countries (LMICs) bear the brunt of this impact, contributing to approximately 77% of all NCD-related deaths and 85% of premature deaths (aged 30–69 years) [2].

Non-communicable diseases (NCDs) represent a significant burden that disproportionately impacts impoverished communities, a phenomenon that is notably more pronounced in low-to-middle income countries (LMICs) [4] due to the double burden of infectious disease and weak health care system [5, 6]. Four modifiable lifestyle risk factors – tobacco use, harmful alcohol consumption, physical inactivity, and unhealthy diet – are primary contributors to non-communicable diseases (NCDs). Tobacco use alone accounts for over 7.2 million deaths annually [7]. Excess salt or sodium intake is responsible for 4.1 million deaths, while harmful alcohol use and insufficient physical activity are attributed to 3.3 million and 1.6 million deaths respectively [8]. These lifestyle risk factors are associated with metabolic risk factors such as overweight and obesity, elevated blood pressure, and raised blood glucose, which contribute to the development of NCDs [7, 8]. Thus, global strategies to control NCDs focus on addressing these modifiable lifestyle factors [2].

The sustainable development goal (SDG) target 3.4 is aimed at reducing NCD-related premature death by one-third by 2030 [9]. In alignment with this goal, Ethiopia set targets to reduce NCD-related premature deaths by 12.5% [10]. Although deaths attributed to NCDs in Ethiopia decreased by 37% between 1990 and 2015, there is limited knowledge regarding the burden of unhealthy lifestyle factors and their association with socio-demographic and metabolic factors [11]. This suggests that national strategies require full implementation of interventions and need to prioritize people with unhealthy lifestyle factors associated with NCDs.

The evidence for the benefit of healthy behaviours on reducing NCDs is well established [12–14]. Multiple lifestyle risk factors are more likely to exhibit a stronger association with NCDs than a single lifestyle-related factor. Hence, it would be particularly beneficial to amalgamate all major all the major lifestyle risk factors to ascertain an individual's overall unhealthy lifestyle. This approach would allow us to identify individuals at significant risk for NCDs and include them in targeted intervention programs [15]. Recent attention has been drawn to the understanding of socio-demographic factors, such as income inequality in relation to unhealthy lifestyles [16–18]. Prioritizing non-communicable disease (NCD) prevention interventions towards socio-demographic groups with multiple unhealthy lifestyle factors could be a cost-effective strategy [19, 20]. However, to our knowledge, no study has shown the socio-demographic correlates of multiple unhealthy lifestyles in Ethiopia. Therefore, this study aims to determine the prevalence of unhealthy lifestyles and their association with socio-demographic groups. Additionally, we also evaluated the association of unhealthy lifestyles with diabetes and hypertension.

## Methods

### Data source

Individual-level data collected from a community-based cross-sectional survey were used. The survey was conducted in 2015 by the Ethiopian Public Health Institute in collaboration with the Federal Ethiopian Ministry of Health (FMOH) and World Health Organization (WHO) using the NCD STEPS instrument [21]. The STEPS survey methods are described in detail elsewhere [22]. Briefly, the survey was conducted among 9, 800 adolescents and adults aged between 15 and 69 years. The survey contains information about the socio-demographic and behavioural characteristics (STEP I); physical measurements for blood pressure, overweight, and obesity were calculated (STEP II); and included biochemical measurement for diabetes, raised blood glucose, and abnormal lipid level (STEP III) (Supplementary information).

### Sociodemographic characteristics

Sociodemographic data such as age (15 – 29, 30 – 44, 45 – 59, and 60 – 69 years), sex, residence (urban or rural), education (no formal schooling, primary school completed, secondary school completed, and college/University completed), marital status (single, married, and common-law), and income (≤ 12,000, 12,000–23, 299,  ≥ 23,300 Birr) were assessed. Participants were recruited from all administrative regions of Ethiopia.

### Unhealthy lifestyle score

Based on previous studies [23], insufficient physical activity, tobacco use, excessive intake of alcohol, and inadequate serving of fruit and vegetable intake, and overweight/obesity were used to construct unhealthy lifestyle scores as an outcome variable.

#### Insufficient physical activity

Physical activity was assessed based on the total time spent on physical activity per day at work, including transport and recreational settings. It was measured using the metabolic equivalent time (MET) in minutes per week spent in physical activity. According to the WHO recommendation on physical activity, performing an equivalent combination of moderate- and vigorous-intensity physical activity below 600 MET-minutes per week was considered as insufficient physical activity [24].

#### Excessive alcohol intake

Alcohol consumption was measured in terms of current and previous drinking (i.e., ever or within 12 months of the interview period) using the concept of a standard drink i.e., any drink containing about 10 g of pure alcohol. Men who reported four or more standard units per day and women who reported three and more standard units per day “ever” or within the last 12 months were classified as having excessive use of alcohol [25].

#### Current smoking

Tobacco use was assessed in terms of current and previous smoking status, duration of smoking, the quantity of tobacco use, smokeless tobacco use, and exposure to second-hand smoking. Respondents who replied, ‘Yes’ for the question “do you smoke cigarettes (any tobacco product)?” were categorized as “current tobacco users”.

#### Dietary intake

Fruit and vegetable intake were used as a surrogate variable for overall dietary quality as there was no comprehensive data on dietary intake. Consumption of fruit and vegetables was assessed in terms of the number of servings, with a serving being equal to 400 g [26]. Participants who reported consumptions of less than five servings of fruit or vegetables per day during the last 30 days of the interview were considered as having a suboptimal diet [27].

### Metabolic factors

Overweight/obesity: a respondent's height (cm) and weight (kg) measurements were taken to calculate body mass index (BMI). BMI was calculated as the respondent's weight in kilograms divided by the square of the respondent's height in meters (kg/m2). Participants with BMI 25–29.9 and  ≥ 30 kg/m2 were classified as overweight and obese, respectively.

#### Diabetes

Blood glucose measurements were taken to assess diabetes. Diabetes was defined as fasting plasma glucose value: >  = 7.0 mmol/L (126 mg/dl) or currently taking medication/s for diabetes.

#### High blood pressure

Both systolic (SBP) and diastolic (DBP) blood pressure were measured three times and the mean values were taken for this analysis. High blood pressure was defined as SBP >  = 140 mmHg and/or DBP >  = 90, or currently taking medication for high blood pressure.

Unhealthy lifestyle scores for each participant were calculated as the sum of the five distinct factors. The potential scores ranged from 0, representing the lowest degree of unhealthy behavior, to 5, indicating the highest. These high scores reflect a significant co-occurrence of risk factors. We defined an unhealthy lifestyle as the presence of three or more risk factors. Given that approximately three-quarters of the survey respondents hailed from rural areas of Ethiopia, a substantial number of participants likely harbored at least two unhealthy lifestyle risk factors. This is because of the fact that consumption of a homemade, beer-like traditional alcohol known as 'Tela' is prevalent in rural areas, coupled with the typically low levels of nutritional knowledge and dietary diversity among rural inhabitants [28].

### Statistical analysis

Demographic characteristics were analysed by examining the proportion of categorical variables, mean and standard deviation (SD) for continuous and symmetrically distributed data, and median and interquartile ranges (25th and 75th percentiles) for continuous and asymmetric data. Bivariable and multivariable logistic regression models were used to determine the association of socio-demographic factors with unhealthy lifestyle using crude and adjusted odds ratio (OR) and 95% confidence interval (CI). When estimating the adjusted OR for sociodemographic factors, we accounted for metabolic factors, including diabetes and high blood pressure. A *p*-value < 0.05 is considered statistically significant for the association between socio-demographic factors and co-occurrence of unhealthy NCD lifestyle risk factors. Chi-square test was conducted to assess the association between unhealthy lifestyle as exposure and diabetes and hypertension as outcomes (separately). Data management and analysis were performed using Stata version 16 (StataCorp LLC, College Station, TX) and R (version 3.6.3, R Foundation for Statistical Computing, Vienna, Austria).

## Results

Of the analysis included a total of 9,800 participants, with a response rate of 95.5%. Three in five participants were females (59.4%), 78.8% did not attend formal schooling and 72.6% were rural residents. The median age of the study participants was 32 years (25^th^ and 75^th^ percentile: 25, 44 y). Two-thirds of the participants (66.0%) had insufficient physical activity per week, 15.3% were classified as they drank excess alcohol per day based on their response and 5.4% of participants were current smokers. Almost all had inadequate fruit and vegetable intake (98.2%) and 10% were overweight/obese. There were significant differences in the prevalence of almost all unhealthy behaviours across sociodemographic characteristics (Table 1).

**Table 1:** Socio-demographic characteristics of the study participant and unhealthy lifestyle factors in Ethiopia

			Unhealthy lifestyle factors			
Variables		Insufficient physical activity	Excessive alcohol intake	Current smoking	Low fruit & vegetable intake	Overweight and Obesity
	**Total sample n (%)**	6,012 (66.0)	1,441 (15.3)	533 (5.4)	9,524 (98.2)	938 (10.1)
*Residence (N* = *9,800)*		*p* = *0.023*	*p* = *0.01*	*p* = *0.00*	*p* = *0.76*	*p* < *0.00*
Rural	7,113 (72.7)	4,428 (65.3)	1,099 (16.0)	421 (6.0)	6,923 (98.0)	322 (4.9)
*Sex (N* = *9,800)*		*p* < 0.00	*p* < 0.00	*p* < 0.00	*p* = 0.08 ()	*p* < 0.00
Female	5,823 (59.4)	3,618 (68)	498 (9.0)	79 (1.4)	5,640 (98.0)	686 (12.8)
*Age groups in years (N* = *9,800)*		*p* < *0.001*	*p* < *0.00*	*p* < *0.00*	*p* = *0.28*	*p* < *0.00*
15 – 29	3,959 (40.4)	2,379 (63.6)	441 (11.6)	140 (3.5)	3,860 (98.3)	264 (6.7)
30 – 44	3,499 (35.7)	2,188 (66.6)	570 (17)	209 (6.0)	3,388 (98)	378 (11.4)
45 – 59	1,690 (17.2)	1,065 (69.0)	327 (20.3)	133 (8.0)	1,640 (98.2)	238 (14.4)
60 – 69	652 (6.7)	380 (70.2)	103 (16.6)	51 (8.0)	636 (99.1)	76 (11.8)
*Marital status (N* = *9,799)*		*p* < *0.00*	*p* < *0.00*	*p* = *0.00*	*p* = *0.09*	*p* < *0.00*
Single	1,705 (17.4)	966 (60.7)	198 (12.3)	69 (4.0)	1671 (98.8)	126 (7.6)
Married	6,593 (67.3)	4,143 (67.0)	1,053 (16.5)	400 (6.1)	6,404 (98.1)	616 (10.0)
common-law*	1,501 (15.3)	903 (67.8)	190 (13.3)	64 (4.3)	1,449 (98.4)	196 (13.5)
*Highest level of education (N* = *9,800)*		*p* < *0.00*	*p* = *0.07*	*p* = *0.65*	*p* = *0.00*	*p* < *0.00*
No formal Schooling	7,661 (78.2)	4,808 (67.0)	1,147 (15.5)	406 (5.3)	7,641 (98.5)	604 (8.3)
Primary school completed	975 (10.0)	579 (65.0)	124 (13.6)	59 (6.1)	941 (97.3)	104 (11.3)
Secondary school completed	653 (6.7)	372 (63.0)	82 (13.6)	40 (6.1)	631 (97.8)	120 (19.0)
College/University completed^♦^	511 (5.2)	253 (55.0)	88 (18.3)	28 (5.5)	491 (97.0)	110 (22.8)
*Annual income (N* = *6,636)*		*p* < *0.00*	*p* < *0.00*	*p* = *0.65*	*p* = *0.00*	*p* < *0.00*
< = 12,000 Birr	4,494 (67.7)	2,978 (71.2)	525 (12.1)	232 (5.2)	4,320 (97.4)	399 (9.4)
12,000 to 23, 299	1,017 (15.3)	634 (65.6)	214 (21.8)	48 (4.7)	1,003 (99.2)	112 (11.5)
23,300 and above	1,125 (17.0)	661 (64.7)	184 (17.3)	63 (5.6)	1,084 (97.2)	213 (20.1)
*Regions (N* = *9,800)*		*p* < *0.00*	*p* < *0.00*	*p* < *0.00*	*-*	*-*
Addis Ababa	815 (6.7)	464 (75.2)	105 (15.0)	28 (3.4)	771 (98.3)	259 (33.0)
Afar	384 (2.1)	284 (74.3)	3 (0.8)	36 (9.4)	383 (99.7)	20 (5.8)
Amhara	1,867 (10.6)	1,064 (59.5)	479 (26.8)	34 (1.8)	1,861 (99.9)	96 (5.3)
B. Gumuz	384 (2.0)	263 (71.1)	105 (28.1)	20 (5.2)	379 (99.2)	25 (6.9)
Dire Dawa	257 (1.6)	164 (73.7)	7 (2.7)	32 (12.5)	254 (99.2)	29 (12.3)
Gambela	295 (3.0)	103 (36.4)	75 (26)	65 (22)	290 (99.3)	27 (9.4)
Harari	214 (2.2)	84 (70)	0 (0)	35 (16.4)	198 (100)	25 (12.2)
Oromia	2,308 (23.5)	1,561 (70)	233 (10.5)	130 (5.6)	2,285 (99.3)	191 (8.7)
SNNPR	1,706 (17.4)	1,079 (66)	196 (11.8)	62 (3.6)	1,552 (92.7)	137 (8.6)
Somali	615 (6.3)	255 (47.1)	14 (2.3)	89 (14.5)	601 (98.4)	84 (14.6)
Tigray	955 (9.7)	691 (75.8)	224 (24.5)	2 (0.2)	950 (99.8)	45 (5.0)
*Diabetes (N* = *8,790)*		*p* = *0.75*	*p* = *0.05*	*p* = *0.048*	*-*	*p* < *0.00*
Yes	244 (2.8)	138 (67.0)	24 (10.4)	20 (8.2)	235 (98.3)	62 (25.9)
*Hypertension (N* = *9,675)*		*p* = *0.91*	*p* = *0.00*	*p* = *0.75*	*p* = *0.95*	*P* < *0.00*
** SBP** > = 140 or **DBP** > = 90	1,871 (19.3)	1,115 (66.1)	315 (17.8)	99 (5.3)	1,818 (98.3)	383 (20.7)

♦Postgraduate completed (*n* = 12)

^*^common-law (Separated = 386, Divorced = 402, widowed = 669, cohabitating = 41, Refused = 3)

SBP >  = 140/DBP >  = 90; diabetes (Yes: FBG >  = 110)

*FBG* Fasting blood glucose, *SBP* Systolic blood pressure, *DBP* Diastolic blood pressure

### Co-occurrences of unhealthy lifestyles

**Fig. 1:**
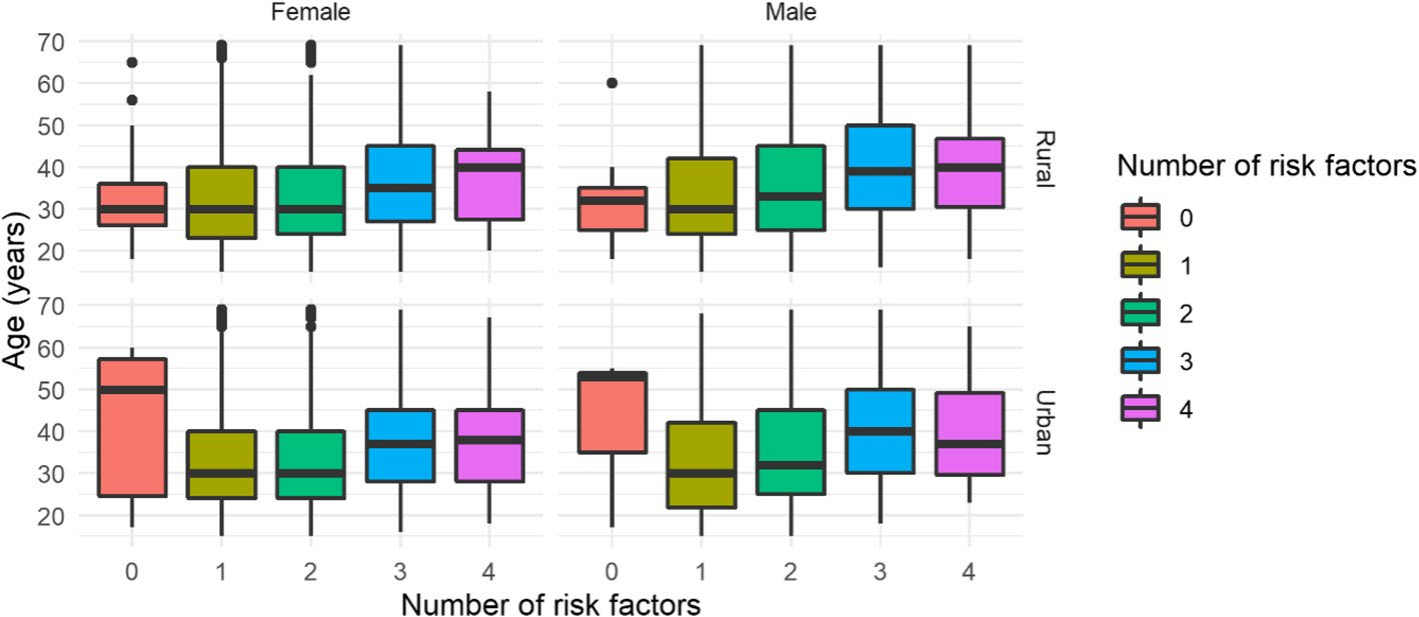
Distributions of unhealthy lifestyle scores across age by sex and residence of the study participants in Ethiopia

**Fig. 2:**
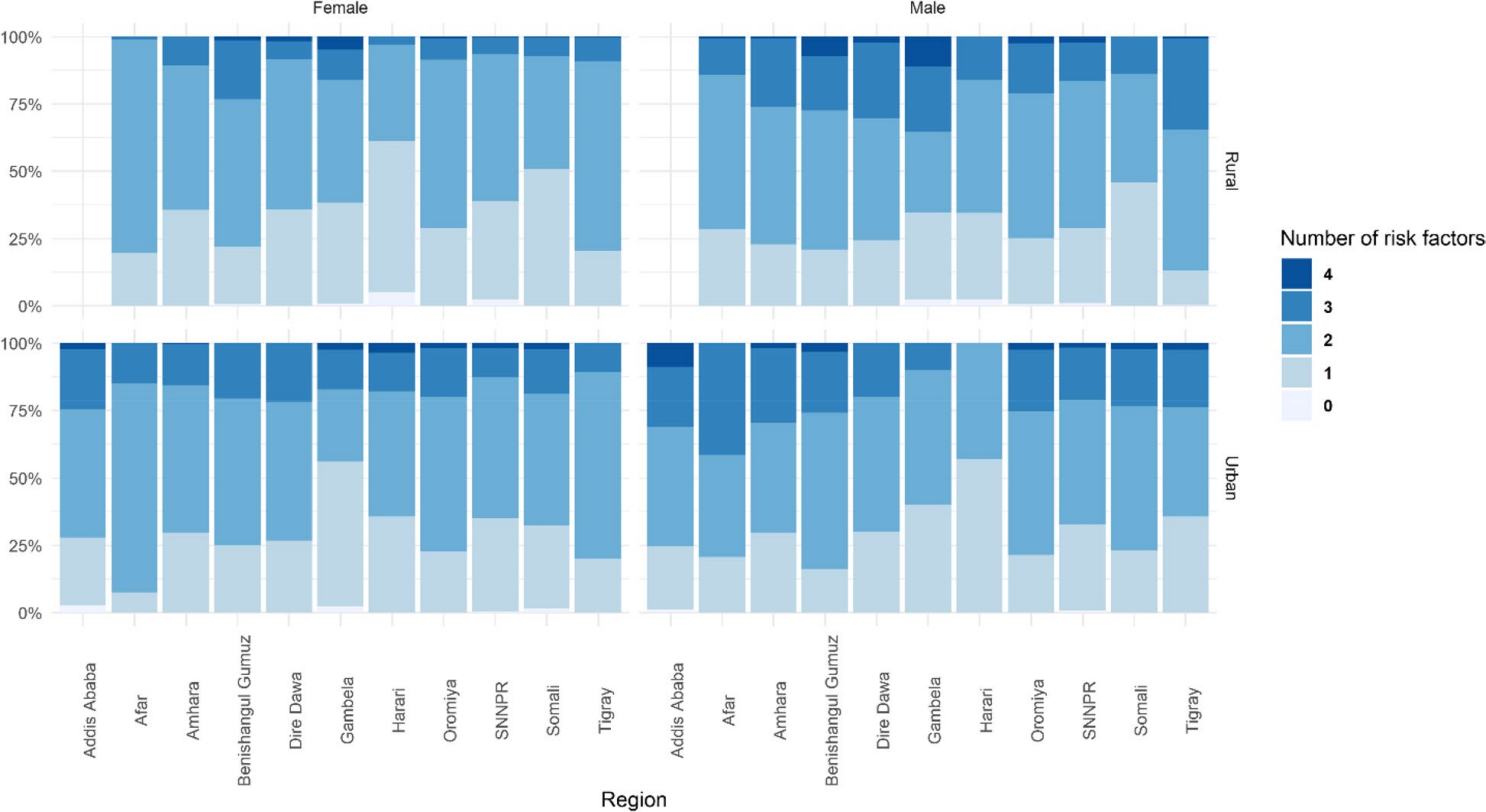
Distributions of unhealthy lifestyle scores of participants in each region by sex and residence of the study participants in Ethiopia. Note: Addis Ababa has no rural villages

Table 2 shows sociodemographic characteristics across unhealthy lifestyle scores. The median unhealthy lifestyle score was three. Only three participants had five risk factors (we added to those who had four risk factors due to the small sample size). One in six participants (16.7%, 95% CI: 16.01, 17.58) had three or more unhealthy lifestyle factors. Significant differences were observed in unhealthy lifestyle scores across various sociodemographic categories. A higher unhealthy lifestyle score was common in older age rural participants (Fig. 1). In most regional administration areas, participants had a least 2 unhealthy lifestyle factors (Fig. 2).

**Table 2:** The numbers of unhealthy lifestyles and socio-demographic characteristics of the study participants in Ethiopia (*N* = 9,796)

		**Number of risk factors**				
		None	One	Two	Three	Four
Variables	n (%)	69 (0.8)	2,806 (32.0)	5274 (55.0)	1,501 (12.0)	147 (0.7)
*Residence (N* = *9,797)*						
Rural	7,111(72.7)	44 (0.6)	2,019 (3)	3,801 (55.1)	953 (13.8)	85 (1.2)
*Sex (N* = *9,797)*						
Female	5,821 (59.4)	49 (0.8)	1, 788 (30.7)	3, 273 (56.2)	659 (11.3)	52 (0.9)
*Age groups in year (N* = *9,797)*						
15 – 29 years	3,959 (40.4)	24 (0.6)	1,272 (32.1)	2,30 (564)	392 (9.9)	39 (1.0)
30 – 44 years	3,499 (35.7)	24 (0.7)	940 (26.9)	1,865 (53.3)	614 (17.6)	55 (1.6)
45 – 59 years	1,690 (17.2)	14 (0.8)	413 (24.4)	846 (50.1)	370 (21.9)	47 (2.8)
60 – 69 years	652 (6.7)	7 (1.1)	181 27.8)	333 (51.1)	125 (19.2)	6 (0.9)
*Marital status (N* = *9,799)*						
Single	1,705 (17.4)	9 (0.5)	573 (33.6)	935 (54.8)	165 (9.7)	23 (1.3)
Married	6,593 (67.3)	42 (0.6)	1,809 (27.4)	3, 523 (55.0)	1,108 (16.8)	109 (1.7)
common-law	1,501 (15.3)	18 (1.2)	424(28.2)	816 (56.1)	228 (15.2)	15 (1.0)
*Highest level of education (N* = *9,797)*						
No formal Schooling	7,661 (78.2)	52 (0.7)	2,156 (30.1)	4,186 (28.1)	1163 (15.2)	102 (1.3)
Primary school completed	975 (10.0)	9 (0.9)	294 (32.3)	516 (30.2)	140 (14.4)	15 (1.5)
Secondary school completed	653 (6.7)	5 (0.8)	190 (32.2)	334 (29.1)	109 (16.7)	15 (2.3)
College/University completed	511 (5.2)	3 (0.6)	166 (40.7)	238 (32.5)	89 (17.4)	615 (2.9)
*Annual income (Birr) (N* = *6,636)*						
< = 12,000 Birr	4,494 (67.7)	40 (0.9)	1,234 (27.5)	2,499 (55.6)	662 (14.7)	59 (1.3
12,000 to 23, 299 Birr	1,017 (15.3)	2 (0.2)	251 (24.7)	552 (54.3)	192 (18.9)	20 (2.0)
23, 300 and above	1,125 (17.0)	10 (0.9)	324 (28.8)	531 (47.2)	224 (19.9)	36 (3.2)
*Region*						
Addis Ababa	814 (8.3)	20 (2.5)	202 (24.8)	381 (46.8)	182 (22.4)	29 (3.6)
Afar	384 (4)	0 (0.0)	84 (21.9)	259 (67.4)	40 (10.4)	1 (0.3)
Amhara	1,866 (19)	1 (0.1)	552 (29.6)	967 (51.8)	337 (18.1)	9 (0.5)
B. Gumuz	383 (4)	1 (0.3)	81 (21.1)	206 (53.8)	81 (21.1)	14 (3.7)
Dire Dawa	257 (2.6)	0 (0.0)	78 (30.4)	132 (51.4)	43 (16.7)	4 (1.6)
Gambela	295 (3)	5 (1.7)	111 (37.6)	108 (36.6)	51 (17.3)	20 (6.8)
Harari	214 (2.2)	7 (3.3)	95 (44.4)	91 (42.5)	20 (9.3)	1 (0.5)
Oromia	2,308 (23.6)	7 (0.3)	599 (26.0)	1, 341 (58.1)	325 (14.1)	36 (1.6)
SNNPR	1,706 (17.4)	25 (1.5)	561 (32.9)	919 (53.9)	178 (10.4)	23 (1.3)
Somali	615 (6.3)	2 (0.3)	266 (43.3)	269 (43.7)	73 (11.9)	5 (0.8)
Tigray	955 (9.7)	1 (0.1)	177 (18.5)	601 (62.9)	171 (17.9)	5 (0.5)
*Diabetes (N* = *8,788)*						
Yes	243 (2.8)	0 (0.0)	59 (24.3)	139 (57.2)	38 (15.6)	7 (2.9)
*Hypertension (N* = *9,675)*						
SBP > = 140 or DBP > = 90	1,871 (19.3)	15 (0.8)	472 (25.2)	946 (50.6)	387 (20.7)	51 (2.7)

### Factors associated with unhealthy lifestyles

Our study demonstrated that males had higher odds of maintaining unhealthy lifestyles compared to females (AOR = 2.27, 95% CI: 1.95, 2.63). Additionally, urban residents were more likely to lead unhealthy lifestyles compared to their rural counterparts (AOR = 1.76, 95% CI = 1.50, 2.06). Notably, the odds of having an unhealthy lifestyle increased with age: participants in the age group of 30–44 years showed higher odds (AOR = 1.66, 95% CI = 1.38, 1.99), those in the 45–59 years age group displayed even higher odds (AOR = 1.99, 95% CI = 1.60, 2.47), and individuals in the 60–69 years age group also demonstrated high odds (AOR = 1.59, 95%CI = 1.18, 2.15) (Table 3).

**Table 3:** Sociodemographic factors associated with unhealthy lifestyle

Variables	At least two unhealthy lifestyle risk factors 8533 (87.0%)	Three or more unhealthy lifestyle risk factors 1264 (13.0%)	COR (95%CI)	AOR (95%CI)
**Residence**				
Rural	5,864 (85.0)	925 (15.0)	1	1
Urban	2,285 (79.0)	338 (21.0)	1.51 (1.35, 1.68) ^**^	1.76 (1.50, 2.06) ^**^
**Sex**				
Female	5,110 (87.8)	711 (12.2)	1	
Male	3,039 (76.4)	937 (23.6)	2.22 (1.99, 2.47) ^**^	2.27 (1.95, 2.63)^**^
**Age (years)**				
15 – 29	3,526 (89.1)	431 (10.9)	1	1
30 – 44	2,829 (80.9)	669 (19.1)	1.93 (1.70, 9.89) ^**^	1.66 (1.38, 1.99) ^**^
45 – 59	1,273 (75.3)	417 (27.7)	2.2.68 (2.31, 12.96) ^**^	1.99 (1.60, 2.47) ^**^
60 – 69	521 (79.9)	131 (20.1)	2.06 (1.66, 6.54) ^**^	1.59 (1.18 2.15)^**^
**Marital status**				
Single	1, 517 (89.0)	188 (11.0)	1	1
Married	5,374 (81.5)	1,217 (18.5)	1.83 (1.55, 2.16) ^**^	1.32 (1.04, 1.68)^*^
common-law	1,258 (83.8)	243 (16.2)	1.56 (1.27, 1.91) ^**^	1.29 (0.94, 1.76)
**Highest level of education**				
No formal Schooling	6,394 (83.5)	1,265 (16.5)	1	1
Primary school completed	819 (84.1)	155 (15.9)	0.96 (0.80, 1.14)	0.98 (0.77, 1.25)
secondary school completed	529 (81.0)	124 (19.0)	1.18 (0.96, 1.45)	0. 95 (0. 72, 1.24)
College/University completed*	407 (79.6)	104 (20.4)	1.29 (1.03, 1.61) ^*^	0.73 (0.55, 0.97) ^*^
**Annual income (Birr)**				
< = 12,000	3,773 (84.0)	721 (16.0)	1	1
12,000 to 23, 299	805 (79.2)	212 (20.8)	1.38 (1.16, 1.63) ^**^	1.46 (1.20, 1.76) ^**^
23, 300 and above	865 (76.9)	260 (23.1)	1.57 (1.34, 1.84) ^**^	1.48 (1.22, 1.79) ^**^
**Diabetes**				
No	7,122 (83.3)	1,423 (16.7)	1	1
Yes	198 (81.5)	45 (18.5)	1.14 (0.81, 1.56)	0.70 (0.46, 1.04)
High blood pressure				
No	6,605 (84.6)	1,199 (15.4)	1	1
Yes	1,433 (76.6)	438 (23.4)	1.68 (1.49, 1.90) ^**^	1.53 (1.30, 1.80) ^**^

*COR* Crude odds ratios, *AOR* Adjusted odds ratios

^*^p-value <0.05

^**^p-value <0.001

Compared to single individuals, being married was associated with higher odds of adopting unhealthy lifestyles (Adjusted Odds Ratio [AOR] = 1.32, 95% CI = 1.04, 1.68). Similarly, individuals with an annual income exceeding 12,000 ETB demonstrated a positive correlation with unhealthy lifestyle choices (AOR = 1.46, 95% CI = 1.20, 1.76 for an income range of 12,000 to 23,299 ETB and AOR = 1.41, 95% CI = 1.12, 1.75 for income of 23,300 ETB and above). However, participants who had achieved a college education or higher presented lower odds of leading an unhealthy lifestyle in comparison to those without formal education (AOR = 0.33, 95% CI = 0.55, 0.97) (Table 3).

### Unhealthy lifestyle factors, diabetes, and hypertension

There were significant associations between excessive alcohol intake and overweight/obesity with diabetes (χ2 = 4.25, *p* < 0.04 for excessive alcohol intake and χ2 = 69.24, *p* < 0.001 for overweight/obesity). Similarly, there were significant associations between excessive alcohol intake and overweight/obesity with high blood pressure (χ2 = 10.30, *p* = 0.001 for excessive alcohol intake and χ2 = 286.78, *p* < 0.001 for overweight/obesity) (Table 4).

**Table 4:** Prevalence (%) and relationships of unhealthy lifestyle factors with diabetes and hypertension in Ethiopia

Unhealthy lifestyle factors	Diabetes		High blood pressure	
	No	Yes	No	Yes
Smoking	*X*^*2*^ = 4.48, df = 1, *p* = *0.034*		*X*^*2*^ = 0.14, df = 1, *p* = 0.71	
No	8, 130 (92.2)	223 (2.5)	7,373 (76.2)	1,772 (18.3)
Yes	441 (5.0)	20 (0.2)	430 (4.4)	99 (1.0)
Excessive alcohol intake	*X*^*2*^ = 4.25, df = 1, *p* = *0.04*		X^2^ = 10.3, df = 1, *p* = *0.001*	
No	6,955 (82.4)	207 (2. 5)	6,409 (69.01)	1,454 (15.6)
Yes	1,259 (15.0)	24 (0.28)	1,109 (11.9)	315 (3.4)
Insufficient physical activity	*X*^*2*^ = 0.151, df = 1, *p* = 0.69		X^2^ = 0.02, df = 1, *p* = 0.88	
No	2,742 (33.5)	68 (0.8)	2,494 (27.7)	572 (6.4)
Yes	5,250 (64.0)	138 (1.7)	4,821 (53.6)	1,115 (12.4)
Low fruit and vegetable intake	*X*^*2*^ = 0.024, df = 1, *p* = 0.88		*X*^*2*^ = 0.004, df = 1, *p* = 0.9	
No	153 (1.8)	4 (0.05)	132 (1.4)	32 (0.3)
Yes	8,308 (95.5)	235	7,594 (79.3)	1,818 (19.0)
Overweight or obesity	*X*^*2*^ = 68.24, df = 1, *p* = < *0.00*		*X*^*2*^ = 286.78, df = 1, *p* = < *0.00*	
No	7,431 (87.8)	177 (2.09)	6879 (74.1)	1,464 (15.8)
Yes	791 (9.3)	62 (0.73)	555 (5.98)	383 (4.1)

## Discussion

In this research, we assessed the socio-demographic determinants of detrimental lifestyle behaviors in Ethiopia, emphasizing tobacco use, heavy alcohol consumption, sedentary behavior, insufficient daily intake of fruits and vegetables, and instances of overweight and obesity. An elevated occurrence of these harmful lifestyle behaviors was observed, with approximately 16.7% of the study population exhibiting at least three of these behaviors. Notably, the prevalence of such lifestyles demonstrated significant associations with variables such as gender, marital status, urban dwelling, advanced age, and a higher income bracket. The findings from this research imply a critical need for targeted health interventions in Ethiopia, focusing on demographics exhibiting a higher prevalence of unhealthy lifestyle behaviors such as urban, older, wealthier individuals and specific gender and marital statuses.

Several large, nationally representative surveys, such as the Global Burden of Disease study, have identified that a significant proportion of NCDs and disability-adjusted life years lost across the globe, including in LMICs are due to mainly modifiable lifestyle factors such as smoking, unhealthy diet, physical inactivity, and inappropriate alcohol consumption [29]. In our study, we found that participants were more likely to engage in two or more unhealthy behaviours. Previous studies [8, 30, 31] have reported similar findings. A high prevalence and co-occurrence of unhealthy lifestyle factors are associated with high burden of morbidity and premature mortality from chronic illnesses, including cardiovascular diseases, diabetes, chronic obstructive pulmonary disease, and some types of cancer [32]. This may eventually lead to healthcare system strain and increased cost of disease management, as well as productivity loss due to illnesses.

Our findings revealed that a high prevalence of co-occurrence of unhealthy lifestyle factors was significantly associated with place of residence. Specifically, participants from urban areas were more likely to have unhealthy lifestyles than their rural counterparts, which is similar with previous studies in Ethiopia [33–35] and other African countries [36–38]. This disparity can be attributed to the fact that urban areas in developing countries, like Ethiopia, are undergoing economic and social developments that contribute to increased exposure to unfavourable environments and behaviours. These behaviours include sedentary lifestyles, smoking, alcohol consumption, and overweight/obesity.

Similarly, our study showed that the likelihood of co-occurrence of risk factors or unhealthy lifestyle increases among participants with higher income, which is in line with previous studies from Ethiopia, Ghana, and Chile. Risk factors, such as overweight and obesity and low physical activity, were associated with wealthier socioeconomic groups [32, 33, 36]. This is a common occurrence in developing countries, where people tend to consume energy-dense and high-fat foods and follow a sedentary lifestyle as their economic condition improves [36, 39]. However, the study showed participants with higher educational status are more likely to engage in physical activity and have a healthier diet. This finding is similar to a study done in Ghana, where Ghanaian adults were more likely to live a healthier lifestyle with increasing levels of educational attainment [36]. This may be because educated people can easily access educational messages on health and risk factors to choose healthier behaviours.

The study also showed variations in the co-existence of unhealthy lifestyle risk factors by socio-demographic factors such as gender, age, and marital status. We observed that despite mixed findings on gender and the number of unhealthy lifestyles, obesity and insufficient physical activity were higher among females than males, while males had higher risks for excessive alcohol intake and smoking [33, 40]. Likewise, our study showed that older participants were more likely to have an unhealthy lifestyle than younger groups. This finding is in line with other similar studies that have found that the prevalence of unhealthy lifestyles, such as smoking, excessive alcohol drinking, and obesity, increases with age [39, 40]. In our study, those who were married also showed a significant association with higher odds of an unhealthy lifestyle. Evidence from Ethiopia and other developing countries indicates that married people are more likely to adopt a sedentary lifestyle [39, 41, 42].

Overall, our study demonstrates and alarming prevalence of co-occurring unhealthy lifestyle factors in Ethiopia, with individual-level risk factors such as excessive alcohol intake, overweight, and obesity being associated with diabetes and high blood pressure. To address these issues, targeted and evidence-based interventions are needed to promote healthy lifestyle habits.

### Implication for policy and practice

Our study examined the co-occurrence of non-communicable diseases in Ethiopia, a country that has undergone rapid socio-economic development and lifestyle changes over the last few decades. While previous studies have documented the prevalence of individual unhealthy lifestyle behaviours, our study is the first to examine how these behaviors co-occur based on sociodemographic characteristics.

Policymakers can use our study as an input to implement comprehensive, policy-level behavioural and public health interventions that promotes healthy lifestyle. By identifying patterns in the co-occurrence of NCD risk factors, our study provides valuable inputs for designing such interventions in Ethiopia. Specifically, our findings highlight the need for context-specific and tailored interventions that take into account sociodemographic factors such as age and sex.

Our study has several strengths, including being the first to examine the co-occurrence unhealthy NCD risk factors and the use of nationwide data collected from a large sample size. However, there are limitations to our findings. One limitation is that the dominant influence of rural areas, due to their larger sample size, may affect the generalizability our results, despite the fact that the prevalence of unhealthy lifestyle issues is primarily observed in urban areas. Additionally, while our study identifies patterns in the co-occurrence of NCD risk factors, further research is commended to determine causality and the effectiveness of interventions.

Overall, our study contributes to a better understanding of the epidemiological transition in Ethiopia and the need for interventions that address the growing burden of NCDs. By designing comprehensive and coordinated interventions that consider the co-occurrence of multiple risk factors, policymakers can help reduce the burden on the country's healthcare system and promote healthier lifestyles for all Ethiopians.

## Conclusion

In our investigation, it was observed that detrimental lifestyle habits are widespread, affecting a significant fraction of the population; approximately one out of every eight participants manifested three or more such habits. We discerned considerable variation in the distribution of these unhealthy lifestyle factors, which was closely tied to sociodemographic attributes. A more advanced level of education correlated with reduced likelihood of poor lifestyle habits. Additionally, conditions such as diabetes and high blood pressure showed a positive correlation with excessive alcohol consumption and overweight/obesity. These observations underscore the imperative for strategic interventions that encourage healthier lifestyle choices, particularly among those with lower educational attainment, as they appear to be at a higher risk. It is crucial that attempts to rectify these detrimental behaviours consider the social and economic factors of individuals to ensure the development and implementation of interventions that successfully tackle the underlying factors of such behaviours.

### Supplementary Information

**Additional file 1.** 

## Data Availability

The datasets used and/or analysed during the current study are available from the corresponding author on reasonable request.
